# Scott Blair Fractional-Type Viscoelastic Behavior of Clear Spruce Wood: Influence of Compression Wood on Power-Law Stiffness Parameters

**DOI:** 10.3390/ma17225477

**Published:** 2024-11-09

**Authors:** Christian Pichler, Roland Maderebner, Alexander Dummer, Thomas Stieb, Roman Lackner

**Affiliations:** 1 Material Technology Innsbruck, Institute of Construction and Material Science, University of Innsbruck, Technikerstraße 13, A-6020 Innsbruck, Austria; roman.lackner@uibk.ac.at; 2 Unit of Timber Engineering, Institute of Construction and Material Science, University of Innsbruck, Technikerstraße 13, A-6020 Innsbruck, Austria; roland.maderebner@uibk.ac.at; 3 Unit of Strength of Materials and Structural Analysis, Department of Basic Sciences in Engineering Sciences, University of Innsbruck, Technikerstraße 13, A-6020 Innsbruck, Austria; alexander.dummer@uibk.ac.at (A.D.); thomas.stieb@uibk.ac.at (T.S.)

**Keywords:** clear spruce wood, basic creep, stiffness, modeling, composition, density

## Abstract

In this paper, we investigate the influence of intrinsic compositional parameters on the viscoelastic compliance by employing three-point bending creep tests on clear, i.e., defect-free, spruce samples with a dimension of 15 × 15 × 280 mm^3^. In addition to the regular samples, a prominent wood variation was investigated: so-called compression wood, stemming from an adaptive response of the growing tree to maintain structural stability. Tests were conducted at constant ambient conditions: isothermal at 20 degrees Celsius and at a relative humidity of 65 percent. These conditions were also employed during sample conditioning, leading to an equilibrium moisture content of the specimens of approximately 12 percent. Hence, so-called basic creep properties were investigated. Furthermore, we show that the experimentally observed compliance can be exceptionally well-modeled by a Scott Blair fractional-type element, with the latter calibrated by a mere number of two independent material parameters. This allows to render rather explicit dependencies of these parameters with respect to the dry density and the volumetric content of the compression wood. There, the quasi-instantaneous stiffness of the employed Scott Blair element is an increasing function of the dry density. While this primary dependency is also observed for compression wood, the quasi-instantaneous stiffness is significantly smaller over the investigated density range.

## 1. Introduction

Reaction wood is a modification of a growing tree caused by a reaction of the stem or branch to handle certain mechanical stresses due to leaning or additional weight [1], leading to altered cell structures with higher density. Reaction wood may be formed in response to sustained bending during growth, e.g., by wind loading on the lee side of stems or by snow loading on the bottom side of branches. In general, reaction wood can be divided into compression wood and tension wood. Whereas tension wood only occurs in hardwood, compression wood is typically found in softwood, such as spruce. When the growing softwood is subjected to pronounced bending, it responds by producing an increased number of wood cells in the compressed region, which may be observed as typical curving of the affected cantilever-like structure, e.g., upward curving of the so-affected branches. The so-formed portion of wood is known as compression wood and is characterized by an altered microstructure and properties as compared to regularly grown wood [2,3,4,5]. One of the most notable features of compression wood is its higher density due to smaller and more tightly packed wood cells. As regards the chemical composition, compression wood is characterized by an increased concentration of lignin, the primary macromolecular constituent as regards strength and stiffness provision in the microheterogeneous cellular composite material. Besides this chemical alteration, compression wood is also characterized by distinctive anatomical features, such as thicker cell walls and smaller lumen diameters, that makes it easily distinguishable in microscopic observations due to its darker, reddish-brown color. Summarizing, the compression wood modification is mainly characterized by (i) thicker cell walls, (ii) a higher lignin content, and (iii) a higher microfibril angle. In turn, these microscopical alternations result in significantly different compositional and mechanical properties [6].

Compared to regular wood, compression wood is characterized by (i) higher density, (ii) lower stiffness and strength, and (iii) increased shrinkage along the longitudinal direction [6,7]. These localized compression wood areas have a gross density that is up to 40% higher. In contrast, the tensile strength is reduced by up to 40% due to the shorter fiber lengths, which increases the risk of brittle fracture. As a result of this undesirable degradation of mechanical properties, limit values for compression wood content must be observed according to various grading methods for use as timber beams or lamellas in structural elements (see, e.g., [8]). Although the effects on quasi-instantaneous strength are well documented in the literature, it is difficult to asses the compression wood content by visual and/or machine methods occurring in industrial processes.

On the other hand, at least to our knowledge, the long-term behavior of compression wood has not yet been investigated. The quantitative assessment of the deflection increase due to creep may be highly relevant for the structural design of timber beams subjected to bending loads. The main aim of this work is to assess the influence of compression wood on the long-term viscoelastic behavior by way of bending experiments. In general, for sustained loads at a moderate level (i.e., significantly smaller than the strength of the material), wood can be assumed as an orthotropic, linear viscoelastic material [9]. Note that the behavior related to high load levels, with modeling according to elastoplasticity, viscoplasticity, and/or damage theory (see e.g., [10]), is not within the scope of this paper; hence, experimental characterization and modeling approaches are not discussed in this introduction.

In the past decades, numerous studies for the determination of the viscoelastic properties of wood have been conducted. For determining the orthotropic viscoelastic behavior of wood in a macroscopic sense, i.e., at the observation scale of engineering application, one may employ creep or relaxation experiments on clear wood specimens. Under the premise of linear viscoelasticity, both types deliver the same outcome, since the compliance function gathered from a creep test and the relaxation function gathered from a relaxation test are inter-related through the Laplace–Carson transform (see, e.g., [11]). Creep experiments on softwood specimens have been carried out under constant environmental conditions in, e.g., uniaxial tension [9,12,13], uniaxial compression [12,13], bending [14], and shear loading [9]. The creep compliance found of wood loaded along the grain direction in tension is significantly lower than in compression. Obviously, the creep compliance as derived from bending tests is expected to be located between the creep compliance in tension and compression, respectively, since bending tests are characterized by both tensile and compressive stresses. A collection of compliance functions gathered from creep experiments can be found in the database by [15]. Alternatively, the viscoelastic characteristics of wood may be assessed by means of a combination of nanoindentation experiments and micromechanical modeling. In this context, the viscoelastic behavior of hot-pressed technical lignin has recently been studied by means of nanoindentation relaxation tests combined with micromechanical modeling by [16]. The time-dependent behavior is significantly influenced by ambient conditions, such as temperature, relative humidity and its corresponding moisture content, as well as changes thereof. Even under constant environmental conditions, i.e., with no heat and no mass (liquid water, water vapor) transport within the sample wood, often referred to as “basic creep”, exhibits significant time-dependent deformation under sustained load (as regards possible thermo-mechano-sorptive coupling effects relevant in creep experiments, see Appendix B). An in-depth assessment and characterization of this behavior may contribute to a more realistic analysis and an improved design of wooden structures. However, since compression wood is generally considered as a deteriorated type of wood and its usage has therefore been restrained, the investigation of its mechanical behavior, in particular the viscoelastic behavior, has received comparatively less attention. Knowledge of its long-term behavior, on the other hand, may foster its structural application, preventing decommission and secondary use.

Only a handful of studies can be found in which the viscoelastic behavior of compression wood has been addressed. Ref. [17] conducted experiments for determining the dependence of short-term creep on the microfibril angle in the secondary cell wall. In this study, compression wood has also been considered as a type of wood with an increased microfibril angle, which was found to substantially increase the creep deformation. More recently, [18] investigated the creep of compression wood fibers in uniaxial tension. They found significantly higher creep deformation of compression wood fibers compared to regular wood fibers. Although there are studies focusing on the characterization of the viscoelastic behavior of compression wood, no studies can be found in which the fraction of compression wood was considered implicitly. All mentioned studies compare compression wood to regular wood, neglecting the fact that, typically, in a specimen, only a certain amount of compression wood is present.

For sustained stresses at a moderate level with respect to strength, wood can be assumed as an orthotropic, linear viscoelastic material [9]. Since the present study is restricted to creep parallel to the grain under constant environmental conditions, the overview of models for the viscoelastic behavior of wood presented here is limited accordingly. Commonly, the viscoelastic behavior of wood is modeled by an assembly of linear springs and dashpots, e.g., Kelvin–Voigt, Maxwell or Burgers models, cf. [19]. Furthermore, a generalized Kelvin–Voigt model has been used for modeling the creep of wood by [20]. Power-law type models have been applied for wood by [9,21]. Note that the listed power-law models can also be interpreted as nonlinear Maxwell models (a Hookean spring in series with a fractional Scott Blair element), for which the relaxation function can be derived analytically from the compliance function, cf. e.g., [11]. Another approach for modeling the creep of wood is the use of logarithmic creep laws, which were employed, e.g., by [22,23]. More recently, [16] found that a Burgers model, i.e., a series coupling of a Maxwell element and a Kelvin element, can well describe the relaxation behavior of hot-pressed technical lignin.

In this paper, we investigate the viscoelastic behavior of clear spruce wood by means of three-point bending creep tests, considering the influence of compression wood on the viscoelastic behavior. Furthermore, we show that a Scott Blair fractional-type element may exceptionally well describe the experimentally observed compliance. In particular, we aim at addressing the following engineering questions:

(i) One may pose the question how the density influences the observed stiffness of compression wood (as compared to the well-described density-based material functions for regular wood)?
(ii) In the case the data are normalized, i.e., when comparing regular wood and compression wood with the same density (but both sample batches are cut from a unique macro-sample, e.g., board), what is the difference in behavior?
(iii) On a more theoretical note, we address the question of which viscoelastic material model may represent the experimental data best, with special emphasis on the medium-term (order of several days) prediction of material behavior.

## 2. Methodology

In this paper, we will use back-calculated material parameters from bending beam experiments and their dependency on dry density and composition in order to address the posed engineering questions.

### 2.1. Preparation and Handling of Specimens

The test specimens were obtained from a total of five tree trunks (in each case the second log of a tree to avoid the influence of the root formation). The clear wood specimens with a length of approximately 280 mm were cut in such a way that the orientation of the tangential plane to the growth rings is parallel to the lateral surfaces. We also ensured that all specimens had a similar annual ring width of 1.5 to 2 mm. The proportion of the compression wood in the cross-section was determined with reflected light microscopy as part of an annual ring width analysis (for photographs of the cross-section of the specimens, see Figure A1). The test specimens were then stored in a standard climate chamber with 20 °C and 65% relative humidity until mass stability.

After conduction of creep experiments, specimens with mass $m_{wet}$ were oven-dried (103 °C) for determination of the dry mass $m_{dry}$ in order to obtain the dry density $\rho =m_{dry}/V$, with *V* denoting the sample volume, and the moisture content as $\left(m_{wet}-m_{dry}\right)/m_{dry}$. Hence, the moisture contents given in this paper reflect the equilibrium state, i.e., mass stability in response to ambient conditions of 20 °C and 65% relative humidity, and amount to approximately 12 m%. The slight variation among samples reflects variations in the sample microstructure and the associated capacity to hold water (also visible in the variation in the dry density among the samples).

### 2.2. Setup of Creep Experiments

For determining the viscoelastic behavior, we employed three-point bending tests on prismatic specimens with a cross-sectional area (b×h) of approximately 15 × 15 mm^2^ and a length of 280 mm [24,25]. Special care was taken as regards alignment of the wood’s microstructural directions (longitudinal, radial, and tangential directions with regard to the growth ring plane) to the specimen directions/coordinates: the wood’s longitudinal material direction coincides with the beam longitudinal direction; the growth ring planes were either parallel to or perpendicular to the lateral surfaces, respectively.

All creep tests were carried out employing the following procedure:

1. Application of the target load with a loading ramp characterized by a constant rate of applied force within a duration of 10 s.
2. Sustaining the target load for a duration of the dwelling phase of 1800 s at a constant temperature level of 20 °C and a sample mass equilibrium associated to an enclosing humidity of 65%, resulting in a moisture content of approximately 12%.
3. Unloading the specimens and subsequently reloading until bending failure within 90±30 s [24,25]. Note that, as the bending strength was determined on specimens which have been exposed to (however, only moderate) loading for the duration of the creep test, the failure strength as reported in this paper does not comply with the prevalent standards for the conduction of strength experiments and needs to be taken with a grain of salt.

During the whole aforementioned procedure, the midspan deflection *u* was monitored by the mean value of two displacement transducers on the front and on the back of the specimen, respectively.

### 2.3. Evaluation of the Uniaxial Compliance Function from Three-Point Bending Experiments

For three-point bending, the elastic solution for the midspan deflection reads

(1) $$u=\frac{Fl^{3}}{48EI},$$

with *F* [N] denoting the midspan load, $I=bh^{3}/12$, and a distance between bearings of *l* = 240 mm. When, on the other hand, investigating viscoelastic material behavior, the history of the midspan deflection u(t) is monitored in response to a step load. To this end, the elastic compliance 1/E is replaced by the uniaxial viscoelastic compliance J(t), i.e., its time-dependent counterpart, in Equation (1), which allows monitoring of

(2) $$J\left(t\right)=u\left(t\right)\frac{48I}{Fl^{3}}.$$

As the underlying instantaneous load application is not possible in real-life experiments, in [11], we coined the term “ramp compliance” for a uniaxial creep test where stress is applied as a ramp with $\sigma \left(t\right)=\sigma_{0}t/t_{0}$, for $0<t<t_{0}$ and held constant afterward as $\bar{J}\left(t\right)=\varepsilon \left(t\right)/\sigma_{0}$ for both the loading and holding phases of the experiment, respectively. Considering here a loading ramp in the bending experiment with $F\left(t\right)=F_{0}t/t_{0}$, for $0\leq t\leq t_{0}$ and held constant, $F=F_{0}$ = const., for $t>t_{0}$, the ramp compliance is given as

(3) $$\bar{J}\left(t\right)=u\left(t\right)\frac{48I}{F_{0}l^{3}}.$$

### 2.4. Constitutive Modeling of Viscoelastic Behavior of Wood with Scott Blair Element

The mathematically consistent form of the compliance function for a Scott Blair element is usually written as

(4) $$J\left(t\right)=\frac{1}{3}\frac{J_{\mathrm{PL}}^{\mathrm{dev}}}{\Gamma [1+n_{\mathrm{PL}}]}{\left(\frac{t}{\tau_{\mathrm{PL}}}\right)}^{n_{\mathrm{PL}}},$$

with material parameters $J_{\mathrm{PL}}^{\mathrm{dev}}$ [${\mathrm{MPa}}^{-1}$], the power-law compliance parameter and $n_{\mathrm{PL}}$ [–], the power-law exponent; $\tau_{\mathrm{PL}}$ [s] is an arbitrarily chosen time constant making the bracket term non-dimensional and was set to 1 s in this paper (for details see [11]). Here, we suppose that the creep process is caused by the deviatoric stress component only; hence, the volumetric creep is neglected. Note that the root-like function for $0<n_{\mathrm{PL}}<1$, with an ever-decreasing creep rate, yields a compliance characterized by a vertical tangent at t=0, i.e., $\dot{J}\left(t\to 0\right)\to \infty$, i.e., there is a quasi-instantaneous response upon a step change in the applied stress depicted by this constitutive element. Further, note that the Scott Blair element reduces to a (deviatoric) Newtonian damper with viscosity of $\tau_{\mathrm{PL}}/J_{\mathrm{PL}}^{\mathrm{dev}}$ for $n_{\mathrm{PL}}$ = 1. For $n_{\mathrm{PL}}\to 0$ a Hookean spring is recovered with Young’s modulus $E=3/J_{\mathrm{PL}}^{\mathrm{dev}}$. In this way, via the exponent $n_{\mathrm{PL}}$, the Scott Blair element is able to represent a continuous spectrum ranging from pure Hookean spring-like behavior to pure Newtonian damper-like behavior; the smaller the exponent, the more spring-like the behavior.

**Figure 1:**
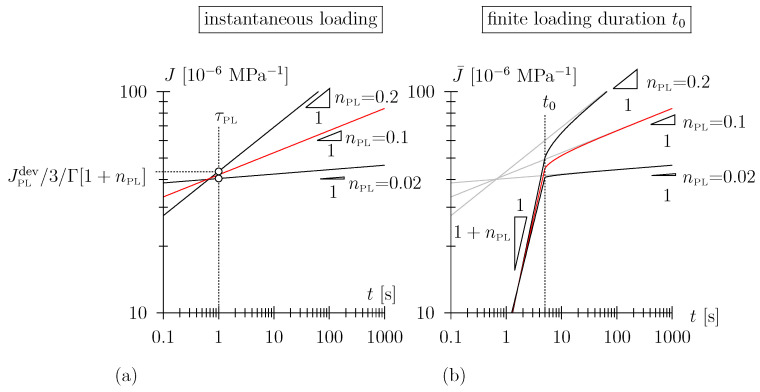
(**a**) Creep compliance J(t), cf. Equation (4) (in log-log scale) of Scott Blair element with $J_{\mathrm{PL}}^{\mathrm{dev}}$ = 120 × $10^{-6}$ ${\mathrm{MPa}}^{-1}$ and $\tau_{PL}$ = 1 s, (**b**) ramp compliance $\bar{J}\left(t\right)$, cf. Equations (A1) and (A2) considering a finite loading duration $t_{0}$ = 5 s (note that in (**b**) the ramp compliance has been superimposed with the compliance function (in gray) for better comparability.)

In [11], we derived analytical expressions for the ramp compliance of a Scott Blair element (see Equations (A1) and (A2) in Appendix A, which will be employed for back-calculation of the material parameters from the experimental data). Reflecting the power-law nature of the Scott Blair element, the natural choice of depiction of the compliance function and the ramp compliance is a log-log diagram, with J(t) characterized by a constant slope of $n_{\mathrm{PL}}$ (see Figure 1a). The ramp compliance $\bar{J}\left(t\right)$ is characterized by a constant gradient of $\left(1+n_{\mathrm{PL}}\right)$ during the loading phase; the long-term gradient of $\bar{J}\left(t\right)$ is given as $n_{\mathrm{PL}}$ (see Figure 1b). In other words: in the case the long-term experimental data linearizes in a log-log diagram, that is a straightforward indication that the viscoelastic behavior is indeed of the Scott Blair type. For small numbers of $n_{\mathrm{PL}}$, e.g., for $n_{\mathrm{PL}}$ = 0.02, in a log-log diagram, the ramp compliance appears almost bilinear (see Figure 1b, though this is not true for the very initial part of the holding phase).

**Figure 2:**
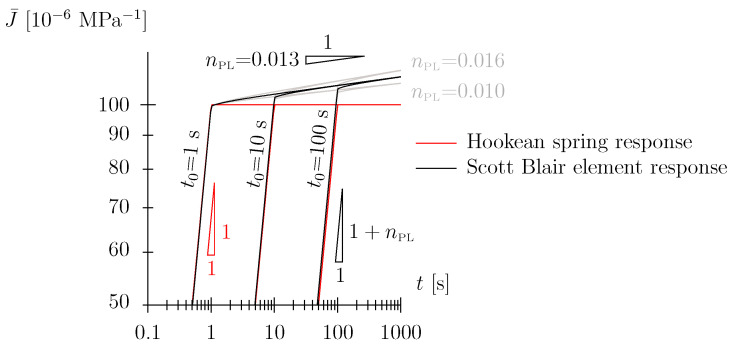
For a Scott Blair element with a small value for $n_{PL}$ the value of $\left(3/J_{\mathrm{PL}}^{\mathrm{dev}}\right)$ is a good proxy for the uniaxial material stiffness, i.e., Young’s modulus *E*. The figure shows the response in three creep tests with different durations of load application $t_{0}$. (red) Hookean spring response, *E* = 10,000 MPa, a ballpark number for the uniaxial stiffness of spruce wood. (black) Response of Scott Blair element with $J_{\mathrm{PL}}^{\mathrm{dev}}$ = 300 × $10^{-6}$ ${\mathrm{MPa}}^{-1}$, $n_{\mathrm{PL}}$ = 0.013 (these are typical numbers obtained in this study).

In this paper, we will show that the back-calculated exponents $n_{\mathrm{PL}}$ for spruce wood (in the investigated density, temperature, and humidity envelope) lie in the range of 0.01 to 0.02. For such low values, the two parameters of the Scott Blair element are rather unambiguously related to the short-term and long-term behavior of the material, respectively. Whereas the long-term viscous compliance increase is represented by the slope of $n_{\mathrm{PL}}$, the quasi-instantaneous, almost Hookean spring-like compliance is approximately given by $J_{\mathrm{PL}}^{\mathrm{dev}}/3$ (see Figure 1b). One may state that for small values of $n_{\mathrm{PL}}$, the value $\left(3/J_{\mathrm{PL}}^{\mathrm{dev}}\right)$ is a good proxy for the material stiffness (in the longitudinal direction), i.e., Young’s modulus *E*. This may be visualized when comparing the response of an elastic material characterized by a certain value for *E* with a Scott Blair material with $J_{\mathrm{PL}}^{\mathrm{dev}}=3/E$ in a creep experiment (see Figure 2). In the case the loading duration $t_{0}$ is sufficiently small, i.e., of the order of a few seconds, the response during the loading phase and the first few seconds of the holding phase does not differ in a substantial manner. Note, recapping, this is only true for small numbers of the power-law exponent, $n_{\mathrm{PL}}\ll 1$, where the behavior of the Scott Blair element becomes Hookean spring-like. These conclusions are not true for larger values of $n_{\mathrm{PL}}$ with a mixed response or when $n_{\mathrm{PL}}$ approaches one with an almost Newtonian damper-like response.

### 2.5. What Is Our Motivation to Abstain from Employing a Hookean Spring in the Viscoelastic Model?

**Figure 3:**
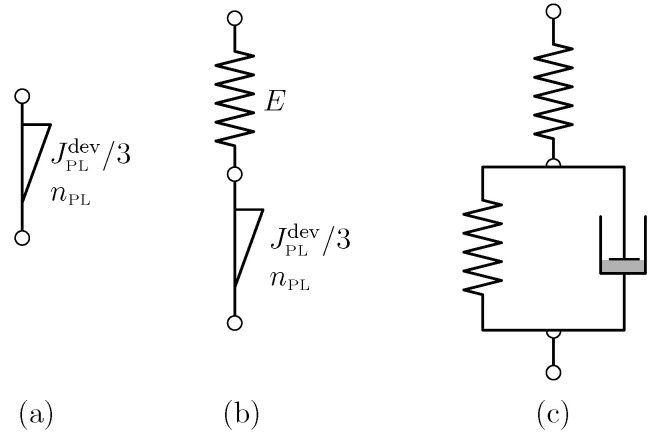
Viscoelastic material models employed in this paper: (**a**) two-parameter Scott Blair element, (**b**) three-parameter nonlinear Maxwell model: Hookean spring connected in series with a Scott Blair element, (**c**) three-parameter Zener model: Hookean spring connected in series with a Kelvin–Voigt element; the latter is constituted by a Hookean spring connected in series with a Newtonian damper.

**Figure 4:**
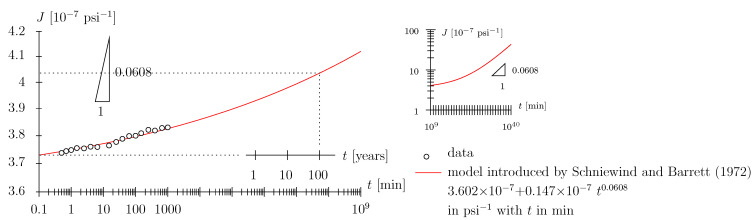
Schniewind and Barrett data [9] in log-log diagram for Douglas fir with moisture content of approximately 10%: board No. 267 data and model for extensional creep compliance (from uniaxial tension experiment) in longitudinal direction with initial value (at *t* = 0.1 min) of 3.73 × $10^{-7}$ ${\mathrm{psi}}^{-1}$; the increase in creep compliance with respect to this value has been extracted from Figure 3 in [9] at times *t* = 0.5, 0.7, 1.0, 1.5, 2.5, 4.0, 6.5, 15, 25, 39, 63, 100, 150, 250, 400, 665, 1000 min as 0.20, 0.38, 0.48, 0.67, 0.64, 0.80, 0.77, 0.92, 1.26, 1.57, 1.84, 1.87, 2.12, 2.45, 2.38, 2.63, 2.69%; note that the long-term prediction (order of years) of the Schniewind and Barrett model may be problematic as the slope (in the log-log diagram) is constantly increasing.

The Schniewind and Barrett data [9] constitute one of the most widely employed data sets for calibration of viscoelastic material models for wood (see, e.g., [26]). The data primarily provide tensile creep data in the longitudinal, radial, and tangential directions, with readings in the range of 0.5 min to 16.7 h, i.e., the very short-term behavior is not accessible (probably due to the limited experimental equipment available in the 1970s). Ref. [9] fitted the data with the so-called nonlinear Maxwell model (see Figure 3b), constituted by a Hookean spring connected in series with a Scott Blair element (see Figure 4, although not of the exact same mathematical form as presented in this paper).

It is possible that lack of data during the loading phase and low resolution data during the beginning of the holding phase may have tempted the authors to depict the quasi-instantaneous behavior with a pure Hookean spring, despite the fact that the initial holding regime (and even the loading regime to some extent) may be characterized by strong nonlinearity caused by viscous behavior.

**Figure 5:**
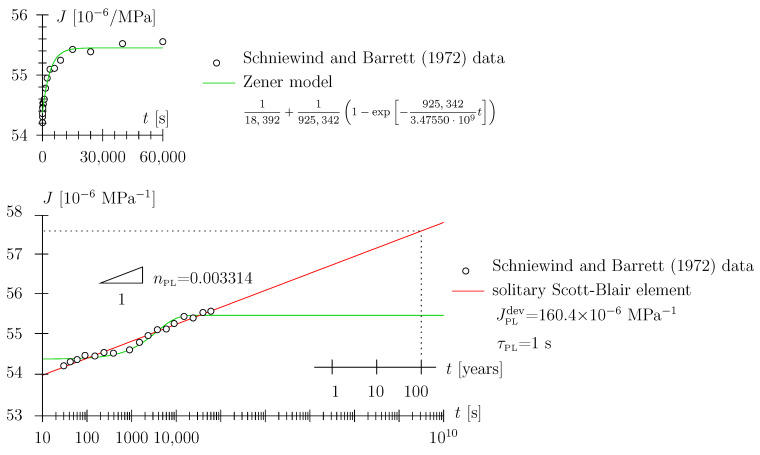
Schniewind and Barrett data [9] in log-log diagram in SI units back-calculated with a solitary Scott Blair element, assuming step load; note that the obtained parameters are much smaller than the ones obtained in the present study: approximately half for $J_{\mathrm{PL}}^{\mathrm{dev}}$ and a quarter for $n_{\mathrm{PL}}$; note that the wood species is different, Douglas fir vs. spruce; the moisture content is smaller, 10%, vs. approximately 12% in the present study; the dry density is 477 kg/m^3^, i.e., right within the range investigated in the present study; further, note that the tensile creep along the grain direction is known to be smaller than the compressive creep, cf. e.g., [1]; hence, the back-calculated parameters are expected to be smaller.

It may seem counter-intuitive to abstain from employing a Hookean spring entirely (this is what we do when employing a solitary Scott Blair element; see Section 2.4 and Figure 1 and Figure 2). However, recall the quasi-Hookean nature of the Scott Blair element for low numbers of the exponent. As regards the use of the nonlinear Maxwell model, one may object that a certain material behavior is assigned in a time range (e.g., zero to 0.5 min) where there is no support from experimental data. With the advent of experimental means allowing detailed monitoring, i.e., high-resolution data logging of the loading phase and the initial holding phase, as will be used in the present paper, there is the possibility to more rigorously assess the quality of the employed viscoelastic models. Based on the Schniewind and Barrett data [9] presented in Figure 4 and Figure 5, one cannot objectively assess which viscoelastic model represents the data in the best way:

- We have already addressed the nonlinear Maxwell model employed by [9] to some extent. An additional point to mention is that the introduction of the Hookean spring leads to a seemingly ever-increasing slope in the log-log diagram; the prescribed power-law exponent of the Scott Blair element $n_{\mathrm{PL}}$ = 0.0608 is reached only after a ridiculously long time span (see small inlay in Figure 4). The allowance of this quasi-ever-increasing slope (in the log-log diagram in terms of engineering application of the model), with the associated implication on the long-term behavior of the order of days or weeks (or even months), i.e., prediction of material behavior, seems, in our estimation, to be too bold a statement to be made based upon the underlying data.
- We might argue that back-calculation of the [9] data with a solitary Scott Blair element (see red graph in Figure 5) may seem appropriate and the prediction of the medium- or long-term behavior quite plausible; however, in fairness, one has to state that, based on data quality, one cannot decide which model depicts reality better. Playing devil’s advocate, this statement may also be tested against other viscoelastic models, e.g., the Zener model: a Hookean spring connected in series with a Kelvin–Voigt element, with the latter constituted by a Hookean spring connected in parallel with a Newtonian damper (see green graph in Figure 5; for a schematic of the Zener model, see Figure 3c). Considering the least-square residual (from the employed nonlinear parameter identification algorithm) as the only measure for model fitness, the Zener model may even be victorious among the models considered. When depicting the data in a linear diagram (see small inlay in Figure 5) one may even trick an unalert reader to think this model is superior to the ones previously discussed. However, considering the (non-existent) long-term predictive capability may constitute a strong motivation for model dismissal.

When one assesses model fitness, one should also ask the question if the model is supposed to depict reality within the time frame of the accessible data or if the future behavior of the material is to be predicted as well, at least to some extent. This questions seems vital as the expected medium- and long-term behavior is of paramount engineering relevance.

The situation is fundamentally different when (i) data quality is better and (ii) loading data and data for the begin of holding phase (i.e., in the vicinity of the sharp bend in Figure 1b) are available from experiments, as is the case in this paper. Note that in the following, we will denote the solitary Scott Blair model as a two-parameter model (parameters $J_{\mathrm{PL}}^{\mathrm{dev}}$ and $n_{\mathrm{PL}}$), and the nonlinear Maxwell (and other related) models as three-parameter models.

### 2.6. Supposition That Three-Parameter Models Are Overdetermined with Respect to the Number of Free Parameters and Lead to Non-Uniqueness of the Back-Calculated Material Parameters

In the case of a complete data set, i.e., including the mentioned sharp bend, we have previously experienced non-uniqueness of the back-calculated parameters for another three-parameter model [23,27]: a Hookean spring connected in series with a Lomnitz element (for a discussion of the latter see [11]). The Lomnitz element is closely related to the Scott Blair element [28], especially in the case of low number exponents. In [23,27], we showed that in this three-parameter model, (i) the back-calculated Hookean spring parameter and (ii) the characteristic time of the Lomnitz element show a mutual dependency. In other words, this three-parameter model is overdetermined with respect to the number of free parameters. When one tries to back-calculate all three material parameters from data in the scope of a nonlinear parameter identification algorithm (Levenberg–Marquardt method [29,30]; the latter is also employed in the present paper), the solution is not unique, i.e., depends on the starting values for the parameters in the algorithm. This may very probably also be true for the nonlinear Maxwell model proposed in [9].

## 3. Results

**Figure 6:**
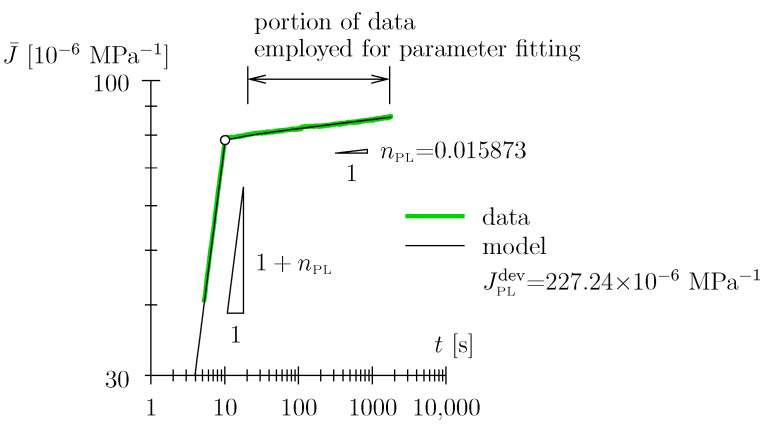
Prototype Scott Blair behavior during the loading and holding phase of the creep experiment for a clear spruce wood sample #2, (ρ = 438.5 kg/m^3^, no compression wood, loading parallel to the growth ring plane).

As regards parameter identification for the Scott Blair element from experimental data, least-squares fitting of the holding phase with the appropriate nonlinear fitting function, i.e., with Equation (A2), was performed for t≥20 s (see Figure 6). Although only the aft part of the data has been employed for the purpose of parameter identification, the entire data range, i.e., also the loading phase (with a slope of $\left(1+n_{\mathrm{PL}}\right)$, see Figure 6) and the beginning of the holding phase are represented with high accuracy by the so-calibrated model response (Equations (A1) and (A2)).

**Figure 7:**
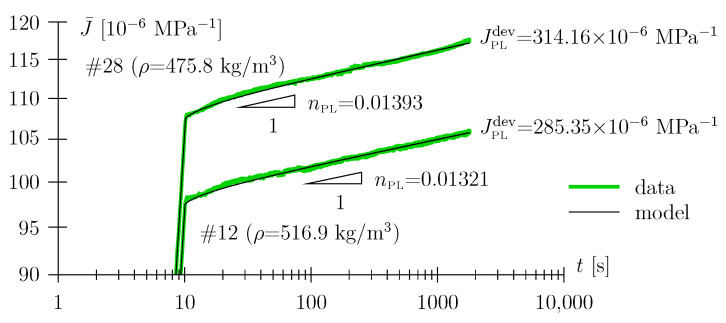
Zoomed-in Scott Blair behavior during the holding phase of creep experiments for clear wood samples #12 and #28 (compression wood content 53% and 44%, respectively; loading parallel to growth ring plane).

This may indicate, besides the fact that the data clearly linearizes in a log-log diagram, that solitary Scott Blair based modeling, with no need for the assignment of a Hookean spring, seems highly appropriate. This is not only true for clear spruce wood (Figure 6), but also for the investigated material modification, i.e., for compression wood (Figure 7).

**Figure 8:**
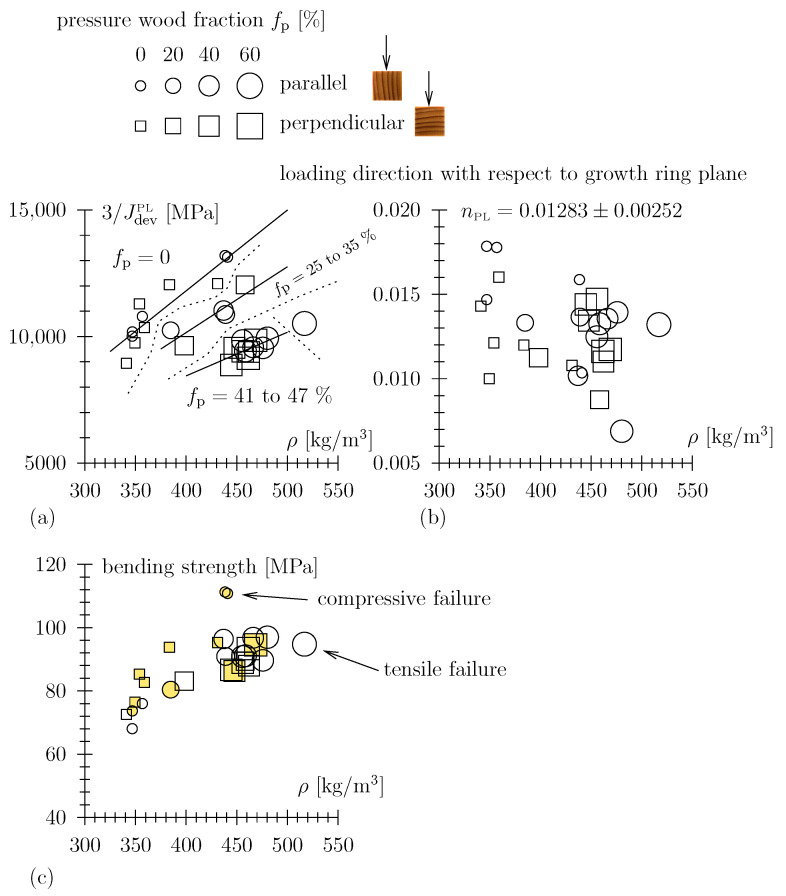
(**a**) Power-law stiffness, (**b**) exponent, and (**c**) bending strength as a function of the dry density, loading direction, and compression wood content, respectively.

The so-obtained material parameters $J_{\mathrm{PL}}^{\mathrm{dev}}$ and $n_{\mathrm{PL}}$, along with the bending strength, as a function of the compositional parameters, along with the experimental parameters, for a total of 28 creep tests, are summarized in Figure 8 and Table 1.

A few notable observations may readily be stated as follows:

- The loading direction with respect to the growth ring plane (either perpendicular or parallel to) does not significantly influence the material parameters (see circle and square symbols in Figure 8). This should come as no surprise as the observed deformation in a slender beam is primarily caused by the normal stress in the longitudinal direction. Geometrical considerations make shear stresses (in the radial or tangential direction, depending on the sample orientation during testing) negligible as regards the observed deformation.
- There is no significant influence of the dry density on the power-law exponent $n_{\mathrm{PL}}$; the latter is back-calculated in a narrow range from approximately 0.010 to 0.015 (see Figure 8b). Some of our unpublished work, however, hints at the possibility that $n_{\mathrm{PL}}$ is a function of the moisture content. As the moisture content has not been varied in the present study (approximately 11 to 12% throughout the experimental campaign), this possible dependency is left to future discussions.

## 4. Discussion

### 4.1. In Reply to Engineering Questions (i) and (ii), Respectively

For a Scott Blair element with a small value for $n_{\mathrm{PL}}$ (the case in our study), the value of $\left(3/J_{\mathrm{PL}}^{\mathrm{dev}}\right)$ is a good proxy for the uniaxial material stiffness (in the longitudinal direction), i.e., Young’s modulus *E* (see Section 2). Based on this argument, let us denote $\left(3/J_{\mathrm{PL}}^{\mathrm{dev}}\right)$ as the “power-law stiffness”, a proxy for Young’s modulus. This power-law stiffness increases with the dry density (or solid volume fraction, i.e., material phases other than porosity in the natural composite material system). In Figure 8a, the size of the symbols refers to the compression wood content. Furthermore, we assorted the data into three categories: (i) no compression wood, (ii) 25 to 35 vol% compression wood, and (iii) 41 to 47 vol% and determined trend lines depicting the dry density dependency for these three categories. Clearly, the dependency of the power-law stiffness on the dry density is also given for the assorted categories. However, the higher the compression wood content, the further this dependency is shifted to lower parts of the figure. This means that when characterizing the intrinsic behavior of the solid material phase in the dry porous material, the compression wood modification is counter-effective as regards the intrinsic stiffness of the solid material phase.

A meaningful explanation for this observation may be found in the altered microstructure of the compression wood. The latter is characterized by a larger microfibril angle in the secondary layer, which is known to degrade the stiffness parallel to the grain even if the density is similar, cf. e.g., [6,31]. In the present investigation, the results for the power-law stiffness $3/J_{\mathrm{PL}}^{\mathrm{dev}}$, which is closely related to the modulus of elasticity, are, therefore, in line with findings in the established literature.

Although the strength properties are not the focus of this paper, specimens were loaded until failure in three-point bending immediately upon completion of the creep experiments. For the sake of completeness, we show these data in Figure 8c. There, the same scaling as observed for the power-law stiffness also applies to the bending strength; the intrinsic behavior of the solid material phase as regards strength is negatively influenced by the compression wood modification.

When considering the mechanical behavior of an entire cross-section of a stem/branch influenced by growth conditions leading to compression wood formation on one side, one may state that the compression wood-modified portion of the cross-section is characterized by a higher density, which may tempt one to assume a somewhat higher stiffness and strength. When, however, breaking down the situation to the solid material phase constituting the porous composite material, the intrinsic stiffness and strength of the solid material phase, which has been influenced by the compression wood growth conditions, is smaller compared to the intrinsic properties of the solid phase in regular growth conditions.

### 4.2. In Reply to Engineering Question (iii)

A solitary Scott Blair element, i.e., when abstaining from employing a Hookean spring in the constitutive model, seems highly appropriate to model the short-term response (order of seconds to hours) of clear spruce wood and seems able to predict, at least, the medium-term behavior (order of days), as the order of hours compliance data clearly linearize in a log-log diagram (constant slope of $n_{\mathrm{PL}}$). Furthermore, for small numbers of the power-law exponent, $n_{\mathrm{PL}}\ll 1$, the quasi-instantaneous response is well represented by the “power-law stiffness” $\left(3/J_{\mathrm{PL}}^{\mathrm{dev}}\right)$ serving as an exceptionally good proxy for the stiffness in the longitudinal direction.

## 5. Conclusions and Outlook

The Scott Blair model was found to exceptionally well describe the experimentally observed compliance in creep experiments on wood, even though this model includes only two parameters, i.e., the power-law compliance parameter and the power-law exponent. In fact, back-calculation of the model parameters on the basis of the discussed creep experiments resulted in a goodness-of-fit probability of 1 (see Appendix C), indicating exceptionally good model fitness. The quality of the presented back-calculation framework benefited to a great extent from the employed analytical expression for the compliance function, capturing, in addition to the load holding phase, the effect of a loading ramp on the observed creep response.

Based on the obtained results, the influence of the dry density and compression wood content, respectively, on the power-law exponent was found to be insignificant. The short-term power-law stiffness (as determined by the compliance parameter), on the other hand, was clearly affected by the compression wood content. According to the results, an increased compression wood content leads to a reduced power-law stiffness, and may be explained by the increased microfibril angle in compression wood. Finally, the improved understanding of the mechanical performance of compression wood paves the way to its use in structural applications, preventing its decommission and secondary use only.

This paper was restricted to the experimental characterization and constitutive modeling of viscoelastic behavior in the grain direction. Currently, we are investigating the behavior perpendicular to the grain direction. This behavior, generally characterized by a significantly higher compliance (as compared to the compliance in the grain direction), is highly relevant for, e.g., the behavior at abutments or localized loading situations in structural timber. Furthermore, in our opinion, the viscoelastic behavior under simultaneous drying/rewetting (mechano-sorptive creep, see Appendix B) is in desperate need of a deep scientific investigation.

## Figures and Tables

**Table 1:** Experimental parameters, material characteristics, and obtained material parameters; experiments with uneven numbers (#1, #3, …) were loaded perpendicular to the growth ring plane ⊥, even numbers (#2, #4, …) were loaded parallel to the growth ring plane ‖.

#	Loading Direction	Midspan Load [N]	Moisture Content [%]	Dry Density [kg/m^3^]	Compression Wood Content [%]	$J_{\mathrm{PL}}^{\mathrm{dev}}$ [10^−6^ MPa^−1^]	*n*_PL_ [–]	Bending Strength [MPa]
1	⊥	225	11.23	341.1	0	335.5	0.01430	72.5
2	‖	225	11.62	438.5	0	227.2	0.01587	111.2
3	⊥	225	11.31	353.9	0	265.8	0.01212	85.3
4	‖	225	11.62	346.8	0	299.7	0.01785	68.1
5	⊥	225	11.54	358.9	0	289.7	0.01602	82.7
6	‖	225	11.19	356.8	0	277.9	0.01778	76.0
7	⊥	225	11.31	349.5	0	307.7	0.01000	76.5
8	‖	225	11.24	440.8	0	228.3	0.01035	110.8
9	⊥	225	11.54	383.5	0	249.0	0.01199	93.8
10	‖	225	11.25	346.9	0	294.7	0.01468	73.7
11	⊥	225	11.36	431.1	0	248.1	0.01078	95.2
16	‖	225	11.93	437.0	35.00	272.1	0.01017	96.4
17	⊥	225	11.62	398.1	32.33	311.2	0.01124	83.1
23	⊥	225	11.84	458.3	30.47	249.1	0.00876	89.3
24	‖	225	11.72	439.0	30.78	275.9	0.01366	90.9
26	‖	225	11.79	384.7	25.11	292.8	0.01332	80.4
14	‖	225	11.91	466.3	41.99	313.3	0.01356	96.8
15	⊥	225	12.04	447.7	41.24	314.2	0.01344	86.3
19	⊥	225	11.81	468.7	48.94	304.5	0.01174	94.6
20	‖	225	12.10	480.2	47.49	302.0	0.00689	97.0
21	⊥	225	11.95	455.9	44.24	318.5	0.01472	88.9
22	‖	225	11.97	458.5	45.54	318.1	0.01325	91.1
25	⊥	225	11.65	462.2	42.11	319.5	0.01101	87.9
28	‖	225	11.91	475.8	44.18	314.2	0.01393	89.6
29	⊥	325	12.00	444.6	44.82	338.2	0.01440	86.7
30	‖	325	11.85	455.7	46.63	305.0	0.01248	90.9
31	⊥	325	11.79	461.4	47.57	328.1	0.01162	93.3
12	‖	225	11.70	516.9	53.27	285.3	0.01321	94.7

**Figure A1:**
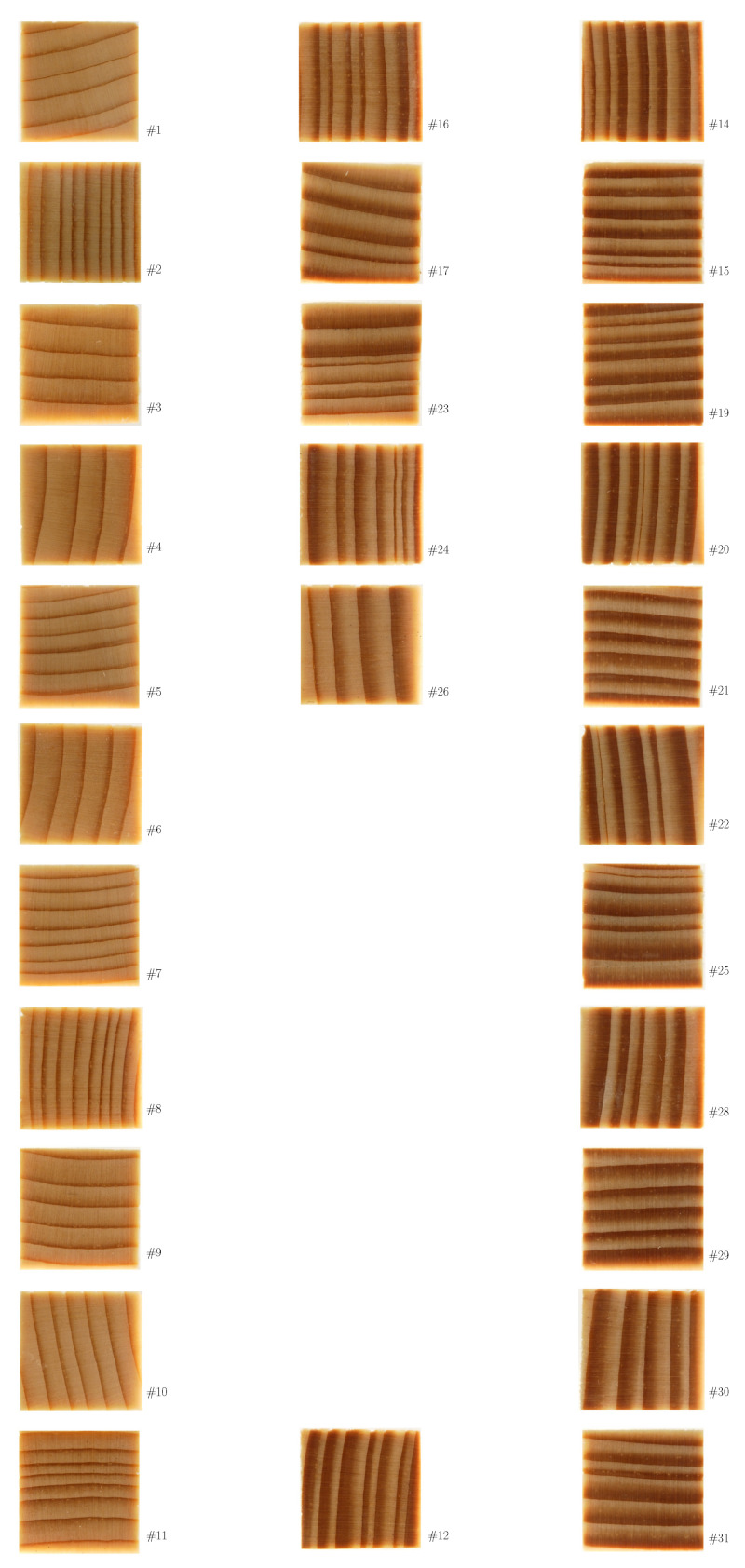
Photographs of cross-section of investigated specimens.

## Data Availability

The original data presented in the study are openly available in Supplementary Materials.

## Supplementary Materials

The following supporting information can be downloaded at: https://www.mdpi.com/article/10.3390/ma17225477/s1.

## Appendix A. Ramp Compliance of a Scott Blair Element

For the Scott Blair element, the ramp compliance is given as

(A1) $$\bar{J}\left(t\leq t_{0}\right)=\frac{1}{3}\frac{J_{\mathrm{PL}}^{\mathrm{dev}}}{\left(1+n_{\mathrm{PL}}\right)\Gamma \left[1+n_{\mathrm{PL}}\right]}\frac{t}{t_{0}}{\left(\frac{t}{\tau_{\mathrm{PL}}}\right)}^{n_{\mathrm{PL}}}$$

and

(A2) $$\bar{J}\left(t\geq t_{0}\right)=\frac{1}{3}\frac{J_{\mathrm{PL}}^{\mathrm{dev}}}{\left(1+n_{\mathrm{PL}}\right)\Gamma \left[1+n_{\mathrm{PL}}\right]}\left[\frac{t}{t_{0}}{\left(\frac{t}{\tau_{\mathrm{PL}}}\right)}^{n_{\mathrm{PL}}}-\frac{t-t_{0}}{t_{0}}{\left(\frac{t-t_{0}}{\tau_{\mathrm{PL}}}\right)}^{n_{\mathrm{PL}}}\right]$$

during the loading and the holding phase of the creep experiment, respectively [11].

## Appendix B. Mechano-Sorptive Coupling Effects Relevant in Creep Experiments

Note that the samples were (i) in thermal equilibrium and (ii) at mass equilibrium associated with the sample enclosing humidity. Hence, possible coupling effects from combined thermo-mechano-sorptive loading/transport processes were circumvented in the experimental program. Such mechano-sorptive coupling effects are known to exist in various engineering materials and, e.g., are termed as the “Pickett effect” in cement-based materials [32] and as mechano-sorptive creep in wood [33]. The Pickett paradox, as summarized in [32], is stated as follows: “A previously dried concrete (sample #1) exhibits practically no creep. In the case of a concrete in which any exchange of moisture during loading is prevented (basic creep), the more evaporable water it contains, the more it creeps (sample #2). On the other hand, a concrete that dries during loading (drying creep) creeps even more (sample #3), although it changes gradually from the hydric state of (sample #2) to the hydric state of (sample #1).” Hence, with regard to creep deformation: (sample #3) > (sample #2) > (sample #1).

## Appendix C. Statistical Analysis of Data

As we have extensively discussed in [34], an apparently acceptable representation of data does not imply that the employed model is fit to depict the physics of the process being measured. This prevalent malpractice may be termed a “chi-by-eye” approach [30], where all too often the coefficient of determination $R^{2}$ is employed to argue for model fitness. Note, however, by no means is the coefficient of determination practicable to objectively justify or reject models. On the other hand, an impartial model check is provided by the goodness-of-fit probability [30]. The goodness-of-fit probability *Q* follows from the chi-square merit function $\chi^{2}$ in the Levenberg–Marquardt algorithm as Q=gammq$\left(\left(N-M\right)/2,\chi^{2}/2\right)$ with gammq as the complement of the incomplete gamma function readily available in [30]. (N−M) is the number of degrees of freedom with *N* as the number of data points and M=2 as two parameters, $J_{\mathrm{PL}}^{\mathrm{dev}}$ and $n_{\mathrm{PL}}$, are fitted. *Q*, with 0≤Q≤1, along with the standard deviations, is given in Table A1 for a measurement error in $\bar{J}$ of $\pm 2\times 10^{-6}$ ${\mathrm{MPa}}^{-1}$. An acceptable model representation is given for *Q* greater than ≈0.1 [30]. Hence, model representation in this paper, with Q=1 for all listed experiments may be characterized as exceptionally good.

**Table A1:** Experimental ID, material parameters with standard deviation, goodness-of-fit probability *Q*.

#	$J_{\mathrm{PL}}^{\mathrm{dev}}$ [$10^{-6}$ ${\mathrm{MPa}}^{-1}$]	*n*_PL_ [–]	*Q* [–]
1	335.50 ± 0.48	0.014308 ± 0.000200	1.0000000000000000
2	227.24 ± 0.48	0.015873 ± 0.000293	1.0000000000000000
3	265.79 ± 0.49	0.012124 ± 0.000256	1.0000000000000000
4	299.66 ± 0.47	0.017845 ± 0.000220	1.0000000000000000
5	289.66 ± 0.47	0.016017 ± 0.000230	1.0000000000000000
6	277.87 ± 0.47	0.017777 ± 0.000237	1.0000000000000000
7	307.68 ± 0.49	0.010005 ± 0.000224	1.0000000000000000
8	228.33 ± 0.49	0.010350 ± 0.000301	1.0000000000000000
9	249.03 ± 0.49	0.011992 ± 0.000274	1.0000000000000000
10	294.67 ± 0.48	0.014686 ± 0.000228	1.0000000000000000
11	248.15 ± 0.49	0.010785 ± 0.000276	1.0000000000000000
16	272.09 ± 0.49	0.010174 ± 0.000253	1.0000000000000000
17	311.15 ± 0.49	0.011241 ± 0.000220	1.0000000000000000
23	249.09 ± 0.49	0.008761 ± 0.000278	1.0000000000000000
24	275.92 ± 0.48	0.013655 ± 0.000244	1.0000000000000000
26	292.81 ± 0.48	0.013320 ± 0.000231	1.0000000000000000
14	313.33 ± 0.48	0.013558 ± 0.000215	1.0000000000000000
15	314.16 ± 0.48	0.013437 ± 0.000215	1.0000000000000000
19	304.51 ± 0.49	0.011739 ± 0.000224	1.0000000000000000
20	301.99 ± 0.50	0.006894 ± 0.000232	1.0000000000000000
21	318.49 ± 0.48	0.014725 ± 0.000210	1.0000000000000000
22	318.10 ± 0.48	0.013254 ± 0.000213	1.0000000000000000
25	319.54 ± 0.49	0.011012 ± 0.000215	1.0000000000000000
28	314.16 ± 0.48	0.013934 ± 0.000215	1.0000000000000000
29	338.20 ± 0.48	0.014405 ± 0.000199	1.0000000000000000
30	305.03 ± 0.48	0.012485 ± 0.000223	1.0000000000000000
31	328.15 ± 0.49	0.011616 ± 0.000208	1.0000000000000000
12	285.35 ± 0.48	0.013207 ± 0.000237	1.0000000000000000
